# Psychometric Properties and Interpretability of PRO-CTCAE^®^ Average Composite Scores as a Summary Metric of Symptomatic Adverse Event Burden

**DOI:** 10.3390/cancers17213459

**Published:** 2025-10-28

**Authors:** Minji K. Lee, Sandra A. Mitchell, Ethan Basch, Allison M. Deal, Blake T. Langlais, Gita Thanarajasingam, Brenda F. Ginos, Lauren Rogak, Tito R. Mendoza, Antonia V. Bennett, Brie N. Noble, Gina L. Mazza, Amylou C. Dueck

**Affiliations:** 1 Department of Quantitative Health Sciences, Mayo Clinic, Rochester, MN 55905, USA; 2 Division of Cancer Control and Population Sciences, National Cancer Institute, Rockville, MD 20850, USA; sandra.mitchell@nih.gov; 3 University of North Carolina Lineberger Comprehensive Cancer Center, Chapel Hill, NC 27599, USA; ebasch@med.unc.edu (E.B.); allison_deal@med.unc.edu (A.M.D.); avbenn@unc.edu (A.V.B.); 4 Department of Quantitative Health Sciences, Mayo Clinic, Scottsdale, AZ 85259, USA; langlais.blake@mayo.edu (B.T.L.); ginos.brenda@mayo.edu (B.F.G.); rogak.lauren@mayo.edu (L.R.); noble.brie@mayo.edu (B.N.N.); mazza.gina@mayo.edu (G.L.M.); dueck.amylou@mayo.edu (A.C.D.); 5 Division of Hematology, Mayo Clinic, Rochester, MN 55905, USA; thanarajasingam.gita@mayo.edu; 6 Center for Cancer Research, National Cancer Institute, Rockville, MD 20850, USA; tito.mendoza@nih.gov

**Keywords:** ACS, total adverse event burden, overall symptomatic adverse event burden, psychometric validation

## Abstract

**Simple Summary:**

This study examined the psychometric properties and interpretability of an average composite score (ACS) as a method of scoring the Patient-Reported Outcome version of the Common Terminology Criteria for Adverse Events (PRO-CTCAE^®^). The ACS is calculated by averaging symptom-level composite scores to create a metric reflecting overall symptomatic adverse event (AE) burden. We analyzed data from patients with breast, lung, or head and neck cancers who were undergoing chemotherapy or radiation therapy. Our goal was to determine whether the ACS is a valid and interpretable summary metric for capturing symptomatic AE burden across different cancer types. We evaluated internal consistency, dimensionality, and model fit using various statistical techniques, including confirmatory factor analysis and principal component analysis. We also used latent profile analysis to explore how well the ACS distinguished patient subgroups with different symptomatic AE profiles. While the ACS provided a summary metric of overall symptomatic AE burden and showed suitable psychometric properties, we also found that patients with similar ACS values had clinically distinct symptom experiences, highlighting the complementary value of both summary scores and detailed symptomatic AE profiles.

**Abstract:**

Background: The PRO-CTCAE provides patient-reported data on symptomatic AEs. A summary metric—the ACS—reflecting total AE burden can be calculated by averaging AE-level composite scores at a given timepoint for each participant. This study investigated the psychometric properties and interpretability of this PRO-CTCAE ACS in patients with breast, lung, or head/neck cancers. Methods: We conducted a secondary analysis of a PRO-CTCAE validation dataset comprising 940 adults undergoing chemotherapy or radiation therapy (clinicaltrials.gov: NCT02158637). We focused on empirically recommended symptom terms for three cancer sites. Analyses included Spearman’s correlations, coefficient alpha, and eigenvalues from the correlation matrices, confirmatory factor analysis (CFA), and principal component analysis (PCA). Latent profile analysis (LPA) was used to assess ACS interpretability in the lung cohort. Results: Mean composite score inter-correlations were moderate (0.30–0.35), and coefficient alphas were high (0.81–0.91). Eigenvalue ratios and CFA supported retention of a single factor/component, with suitable model fit indices. ACS correlated highly with factor scores and the first principal component from the PCA. Reduced sets of terms produced reliable scores that closely approximated the full set scores and aligned with external criteria. LPA in the lung subgroup identified four latent classes; ACS differentiated high vs. low symptom burden groups but did not distinguish the two groups expressing distinct symptom profiles. Conclusion: The ACS demonstrated structural validity through adequately fitting linear factor models and effectively summarized symptomatic AE burden. However, similar ACS values may mask clinically distinct symptomatic AE profiles, underscoring the value of both summary metrics and profile-based approaches.

## 1. Introduction

Cancer patients often endure a substantial symptom burden stemming from their disease and the side effects of anti-cancer treatments. To address this, the National Cancer Institute (NCI) developed the Patient-Reported Outcomes version of the Common Terminology Criteria for Adverse Events (PRO-CTCAE^®^), a measurement system comprising 124 items that assess 78 symptomatic adverse events (AEs) by patient self-report [1]. PRO-CTCAE complements the Common Terminology Criteria for Adverse Events (CTCAE), the standard system for clinician reporting of AEs in cancer clinical trials.

Capturing patient-reported symptomatic AEs is essential towards a comprehensive understanding of treatment impacts, evaluating the balance of benefits and risks, and comparing treatment options [2]. Regulatory authorities, professional organizations, and advocacy groups have recognized the importance of incorporating patient-reported symptomatic AEs into cancer drug development.

Selecting PRO-CTCAE items typically involves considering expected symptomatic AEs based on clinical data and the specific treatment regimen [3]. A common approach is to analyze PRO-CTCAE items by summarizing AEs that are new or have worsened compared to baseline (i.e., baseline adjusted method) [4,5,6], or calculating AE-level composite scores to combine frequency, severity and interference attributes [7]. To facilitate comparisons of AEs between arms, recent efforts have introduced an overall AE burden score using clinician-reported CTCAE data [8]. Similarly, given the extensive number of PRO-CTCAE items, a summary measure is valuable for capturing the overall AE burden reported by patients. A previous study developed a toxicity index that summarizes PRO-CTCAE scores across timepoints for an individual [9]. The integer part of the index reflects the highest score across AE terms and timepoints, while the decimal part captures all other AE experiences beyond the most severe AE. However, the emphasis on the most severe AE in this metric may overshadow less severe but still burdensome AEs, potentially underestimating the cumulative burden of multiple concurrent moderate AEs. To address this, we propose the PRO-CTCAE average composite score (ACS), which calculates the mean of PRO-CTCAE composite scores for each AE term. By averaging across composite scores, the ACS produces a continuous cross-sectional summary score, ranging from 0–3 at each time of assessment. If shown to have robust measurement properties and strong interpretability, the ACS could offer a simple to calculate summary metric reflecting the patient reported overall burden of symptomatic AEs at each assessment timepoint.

In quality-of-life and multi-symptom symptom assessment tools that produce a series of item-level scores, scoring algorithms like sum scores (often called raw scores) or linear transformations of the sum scores (often averages or transformation to a 0–100 metric) have been commonly used to summarize complex constructs into a single value [10,11,12,13,14,15,16,17,18]. These scoring algorithms offer an ordinal approximation of the underlying latent variable across a set of self-reported indicators, for example, symptoms, activity impairments, or concerns [19]. If a linear or nonlinear factor model applies, the sum score exhibits the crucial property of being monotonically related to the latent variable. Using classical test theory, which operates under the assumption that each observed score reflects a combination of the true score and random error, regardless of the questionnaire’s dimensional structure, the sum score or transformed score is treated as an estimate of the true score of the overall construct. This relationship holds irrespective of whether the items measure a single construct or multi-dimensional constructs. Moreover, the sum score and its linear transformation such as the average score are easier to calculate compared to a latent-variable estimate.

The aim of the current study is to evaluate the psychometric properties and interpretability of the ACS, calculated as the mean of PRO-CTCAE composite scores across symptomatic AE terms previously identified as salient for each of three cancer sites (lung, breast, and head/neck) and their treatment approaches. Demonstrating favorable measurement properties of the ACS would support its use as a summative cross-sectional indicator in future studies.

## 2. Materials and Methods

### 2.1. Data

For this secondary analysis, we used PRO-CTCAE data collected in a multi-site validation study [20,21,22,23] of 940 adults who were receiving or initiating chemotherapy and/or radiation therapy at nine U.S. cancer centers or community oncology practices (clinicaltrials.gov: NCT02158637). Using data from this study’s baseline timepoint, we drew three subsamples of patients from the original sample to create a lung cancer cohort (*N* = 183), a breast cancer cohort (*N* = 260), and a head/neck cohort (*N* = 146). A majority of the sample had received treatment with surgery, radiation or chemotherapy within the two weeks prior to enrollment.

### 2.2. Measures

#### 2.2.1. PRO-CTCAE Symptom Terms

Recent mixed-methods studies [24,25,26,27,28,29,30,31,32] identified disease-specific subsets of PRO-CTCAE symptom terms for prospective surveillance of symptomatic toxicities. Our analyses focused on PRO-CTCAE symptom terms empirically identified in patients with lung cancer receiving various treatment modalities (8 terms) [32], breast cancer patients undergoing anticancer drug therapy (16 terms) [28], or head and neck cancer patients receiving radiation therapy (17 terms) [30] (Appendix A: Table A1). Each PRO-CTCAE term was measured by frequency, severity, interference or a combination of these attributes [3]. PRO-CTCAE responses were provided on a 0–4 ordinal scale with verbal descriptors of ‘never’ to ‘almost constantly’, ‘none’ to ‘very severe,’ or ‘not at all’ to ‘very much’. To provide a single representative value per symptom term for this analysis, composite scores [7] were calculated. Composite scores aggregate item-level scores from up to three PRO-CTCAE attributes into one of four ordinal categories, represented numerically by an integer that can range from 0 to 3. To generate composite scores, we used the ‘toxScores’ function in the R package, ‘ProAE’ (version 1.0.3) [33], which processes PRO-CTCAE item responses and returns a dataset with corresponding composite scores. The overall burden of symptomatic AEs was then calculated by averaging across all the AE-level composite scores for each participant to produce the ACS. The ACS ranges from 0 to 3, independent of the number of AE symptom terms.

#### 2.2.2. EORTC QLQ-C30

The EORTC QLQ-C30 was administered concurrently with PRO-CTCAE in this study. In the concurrent validity analysis, we calculated the QLQ-C30 summary score by averaging 13 scales and items, excluding the global health status/quality of life and financial impact scales. All scales and items were coded so that higher scores indicate worse outcomes, aligning with PRO-CTCAE scoring where higher scores indicate greater symptom frequency, severity, and/or interference. To gauge concurrent validity, we explored the relationship between the PRO-CTCAE ACS and the QLQ-C30 summary score. The QLQ-C30 summary score has shown strong prognostic value for overall survival across various cancer populations, outperforming any individual scale within the QLQ-C30 [15].

### 2.3. Handling Non-Administered Items

The dataset used in the present analysis employed a survey administration schedule (as illustrated in eTable 1 of Dueck et al. [20]) that was designed to optimize respondent exposure to PRO-CTCAE items while also conserving participant response burden. This resulted in missing data that was expected by design for some of the PRO-CTCAE composite scores due to non-administered items. Missing-by-design rates ranged from 3% to 7% (mean: 6%) across composite scores in the lung cohort, 11% to 37% (mean: 22%) in the breast cohort, and 3% to 32% (mean: 13%) in the head/neck cohort. Because only 1% of the data was missing across the 15 EORTC-QLQ C30 scales, which were administered across the three cohorts at the same time as PRO-CTCAE, the missing data in PRO-CTCAE composite scores was primarily attributable to non-administered items rather than patient non-responsiveness. This missing data, therefore, likely depends only on observed variables, such as cohort membership and survey administration phase, meeting the assumptions of missing at random and supporting our use of data imputation.

Accordingly, to preserve sample size and avoid listwise deletion when confirmatory factor analyses (CFA) are performed using the weighted least squares estimator, we conducted missing data imputation. For missing data imputation, we used the ‘missForest’ package version 1.5 in R version 4.3.3, which predicts missing values by leveraging the observed relationships among variables, including complex interactions and nonlinearities [34]. The default settings of the ‘missForest’ function were used, with a maximum of 10 iterations and 100 trees grown for each forest. Variables included in the imputation process for each cohort were EORTC QLQ-C30 single- or multi-item subscale scores, PRO-CTCAE composite scores, and demographic and clinical characteristics, such as disease site; age at enrollment; sex; race; ethnicity; receipt of radiation therapy, surgery, and/or chemotherapy, education level, and ECOG performance status.

### 2.4. Analyses

#### 2.4.1. Internal Structure of the PRO-CTCAE Composite Scores

We examined Spearman’s rank correlation coefficients among AE-level composite scores and determined the number of factors (or components) to retain using the eigenvalue ratio test. This approach identifies a sharp drop in variance explained between successive components [35]. For each factor *i*, we also calculated the ratio (*R_i_*) of its eigenvalue *λ_i_* to the eigenvalue of the next factor *λ*_*i*+1_, identifying *i* with the largest ratio. For example, if R_1_ is largest, we retain one component; if *R*_2_ is largest, we retain two components. Based on the examination of the eigenvalue plot and this test, we conducted principal component analyses (PCA) to extract the identified components. The first principal component, PC1, captures the shared variation across scores, providing a simplified summary of their commonalities. We correlated the ACS with PC1 to assess their alignment.

We evaluated one-factor CFA models using diagonally weighted least squares estimation, a method designed for ordinal data [36]. Model fit was assessed using Comparative Fit Index (CFI), Tucker–Lewis Index (TLI), Root Mean Square Error of Approximation (RMSEA), and Standardized Root Mean Square Residual (SRMR) [37]. Acceptable fit criteria were defined as CFI and TLI ≥ 0.95, RMSEA ≤ 0.06 (with the lower limit of the 90% confidence interval including 0.06), and SRMR ≤ 0.08. Additionally, the chi-square (*χ*^2^) statistic, degrees of freedom, and corresponding *p*-value were reported. A *p*-value > 0.05 generally suggests good model fit, and significant *χ*^2^ tests can result from multivariate non-normality or high proportions of unique variance. In addition to the global goodness-of-fit statistics, we examined residual correlations greater than or equal to 0.20 or higher to assess local dependence among composite scores. We evaluated the improvement in model fit allowing for residual correlations between composite scores exhibiting higher correlations. We report unstandardized factor loadings as well as standardized ones. Additionally, we assessed the correlation between the ACS and the latent variable estimate from the confirmatory factor analysis (CFA) model in each cohort. Lastly, we summarized the distribution of the ACS and reported McDonald’s *ω*, a more robust alternative to Coefficient *α*, as it provides a more general and accurate estimation of reliability as it does not rely on the assumption of equal factor loadings (tau-equivalence) [38].

#### 2.4.2. Evidence Based on Relationships to Other Variables

We evaluated the correlation between the ACS and the QLQ-C30 summary score. A strong correlation (≥0.80) with the established criterion such as QLQ-C30 summary score provides evidence of the concurrent validity of the ACS.

#### 2.4.3. Impact of Number of Symptom Terms Used to Calculate the ACS on Reliability and Validity

In certain contexts, trialists may opt to administer only a small number of symptom terms focusing on those most relevant to the anticipated toxicity profile of a given treatment. As such we were interested to explore whether reliability, and structural and concurrent validity would be adversely affected when the ACS was calculated using fewer AE-level composite scores. Using the breast and head/neck cohorts as examples, we examined the impact of sequentially removing terms—starting with those symptom terms that were least endorsed by patients [28,30] and sequentially removing terms until reaching a reduced set of only 3 symptom terms. The projected impact of including fewer symptom terms in the calculation of the ACS was evaluated across several metrics, including the percentage of variance explained by PC1, McDonald’s *ω*, CFA fit indices, correlations between the ACS and PC1, the CFA factor score derived using the reduced set, and correlation between the ACS and QLQ-C30 summary score.

#### 2.4.4. Sensitivity and Clinical Interpretability of ACS in the Lung Cancer Cohort

We used LPA to explore the sensitivity and clinical interpretability of the ACS scores. Using the lung cohort as an example, LPA was performed to identify distinct latent profiles of patients based on their AE-level composite scores and to evaluate the relationships between membership in a latent profile subgroup and the ACS. In LPA, latent class membership is treated as an unobserved categorical variable, and the model estimates the posterior probabilities of an individual belonging to each latent profile subgroup [39]. The analysis was conducted using Mplus version 8.9 [40] using full information maximum likelihood (FIML) estimation. Models with 1 to 6 latent classes were estimated. To evaluate model fit and determine the optimal number of latent classes, we considered a series of statistical indices, including the Bayesian Information Criterion (BIC), with lower values indicating better fit, and entropy, where values nearing 1 indicate better class separation. Additionally, we considered theoretical relevance, clinical interpretability, and latent profile class sample size when determining the final number of latent class profiles, and average posterior probabilities were also examined to assess classification accuracy. After selecting the best fitting latent class profile solution, profile-specific distributions of AE-level composite scores for each AE were plotted to visualize profile differences.

## 3. Results

### 3.1. Sample

Table 1 summarizes participant characteristics at study enrollment. The average age was 61 years in the lung cohort, 54 years in breast, and 56 years in head/neck. The majority were female in the lung (54.1%) and breast (98.5%) cohorts, and male (77.4%) in the head/neck cohort. The sample was diverse with respect to race; non-white participants comprised 18.6%, 34.2%, and 21.2%. Hispanic participants comprised 4.4%, 8.1%, and 5.5% of the lung, breast, and head/neck cohorts, respectively. The sample proportion with an ECOG performance status of 2–4 was 23.5% (lung), 6.2% (breast), and 19.9% (head/neck). At the time of enrollment, 42 patients (23.0%) in the lung cancer cohort, 7 patients (2.7%) in the breast cancer cohort, and 96 patients (65.8%) in the head and neck cancer cohort were undergoing both chemotherapy and radiation therapy. Additionally, 4.9% of lung (9/183), 26.2% of breast (*N* = 68/260), and 1.3% of head and neck patients (*N* = 2/146) had not yet commenced their treatment with chemotherapy, radiation, or surgery at the time of enrollment.

### 3.2. Distribution of the PRO-CTCAE Composite Scores

**Figure 1:**
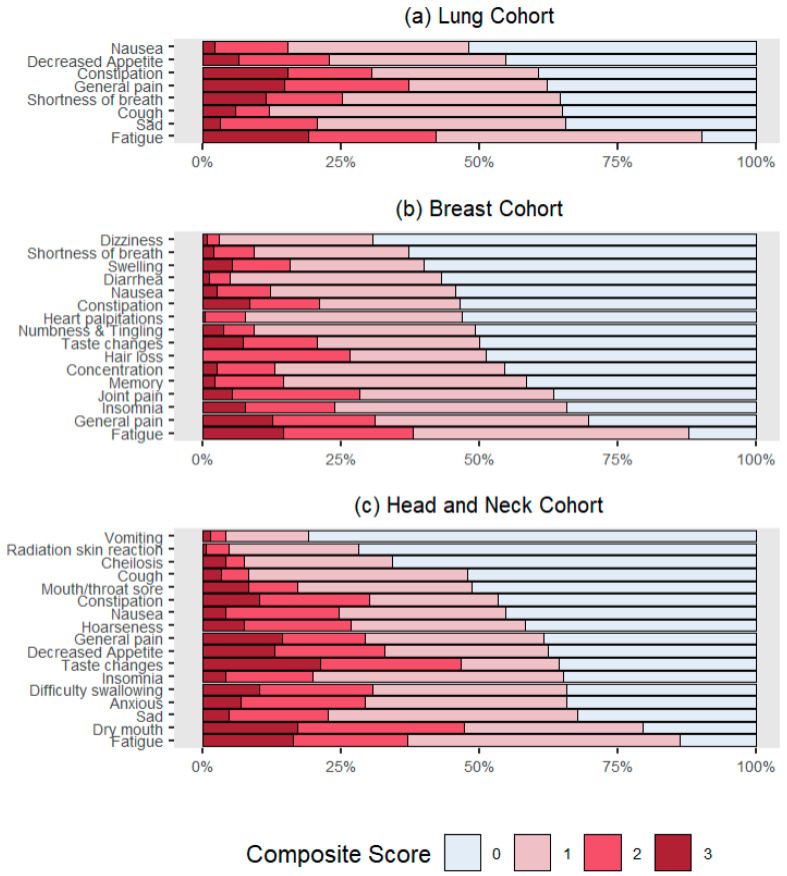
Stacked bar plots illustrating the distribution of composite score categories for selected terms across cohorts. Note. Stacked bar plots illustrate the percentage distribution of composite score categories (0–3) for selected PRO-CTCAE terms across three cancer cohorts: (**a**) Lung, (**b**) Breast, and (**c**) Head and Neck. Higher composite scores reflect greater symptom severity.

Figure 1 shows the distribution of AE-level composite scores across cohorts. In the lung cohort, the most prevalent symptoms included fatigue, cough, shortness of breath, and pain. For the breast cohort, fatigue, pain, insomnia, and aching joints had the highest prevalence. The symptoms with the highest prevalence in the head/neck cohort included fatigue, dry mouth, difficulty swallowing, insomnia, and taste changes. Mood disturbance was reported by 66% of the lung cohort (sadness), and 66% (anxiety) and 68% (sadness) in the head/neck cohorts, respectively.

### 3.3. Structural Validity of the PRO-CTCAE Composite Scores and the ACS

#### 3.3.1. Pairwise Correlations and Principal Component Analyses

**Figure 2:**
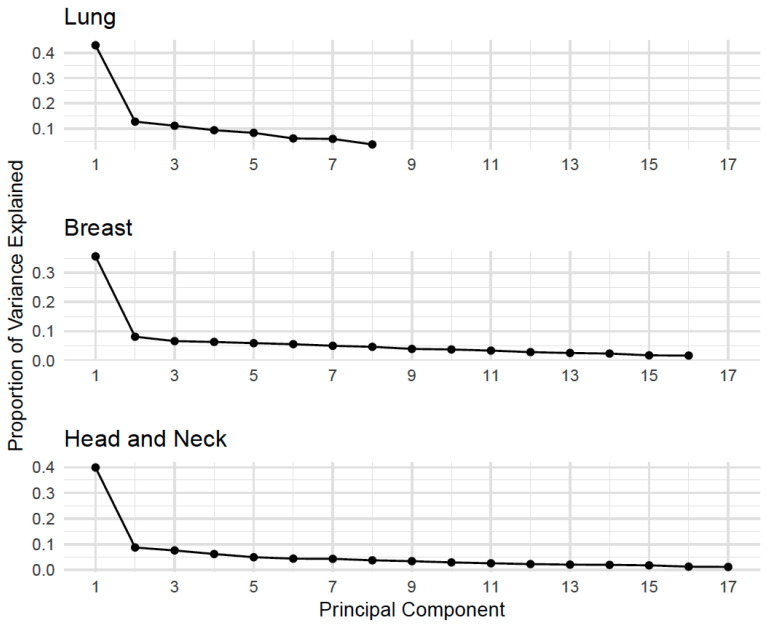
Scree Plots of Principal Components by Cohort: Lung, Breast, and Head and Neck Cancer. Note. Scree plots display the proportion of variance explained by each principal component for each cancer cohort. These plots help identify the number of components that capture the most variation in PRO-CTCAE responses across tumor types, supporting dimensionality reduction and informing subsequent analyses.

**Figure A1:**
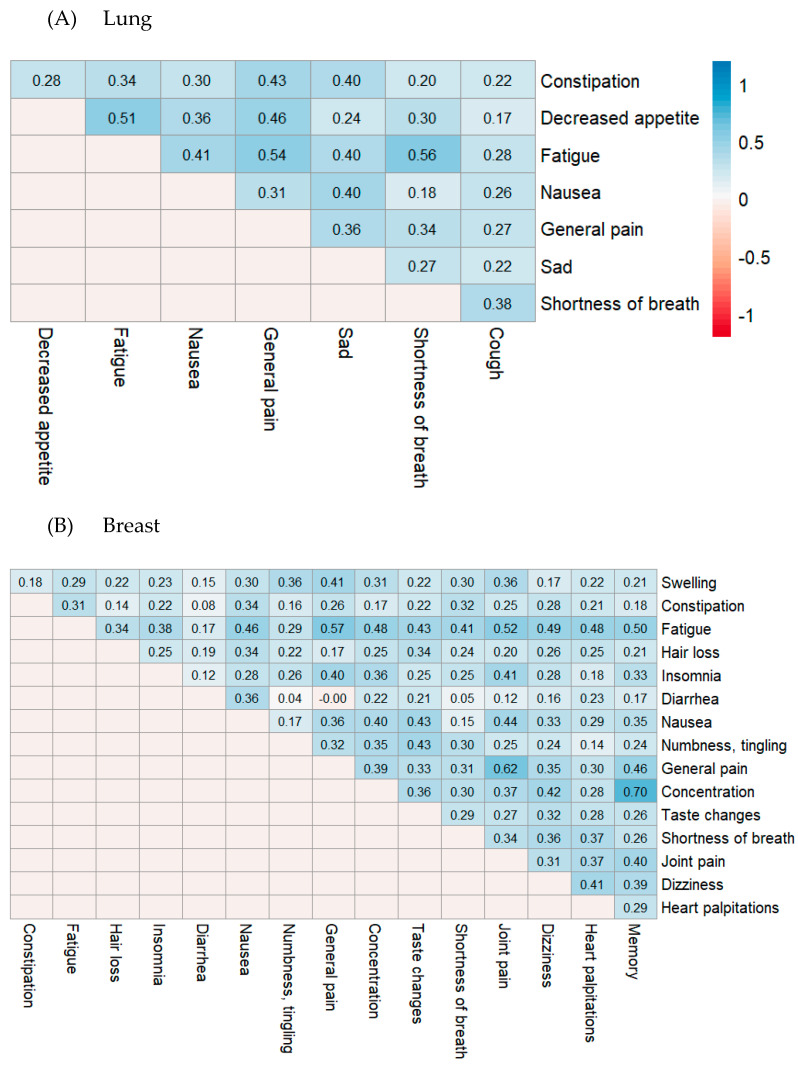

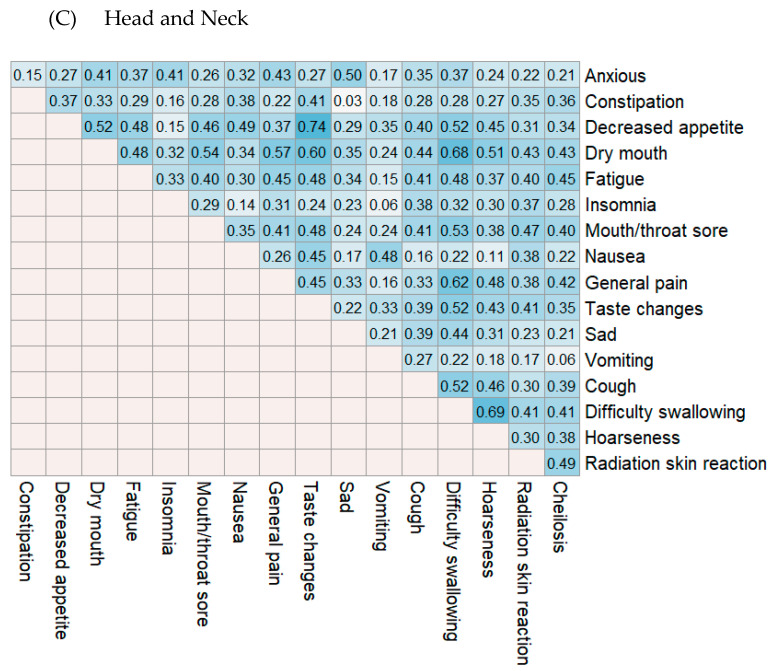
Pairwise Spearman’s Correlations of Composite Scores.

The mean pairwise Spearman’s correlations among the PRO-CTCAE composite scores were 0.34 in the lung cohort (range: 0.17–0.56), 0.30 in the breast cohort (range: −0.00–0.70), and 0.35 in the head/neck cohort (range: 0.03–0.74) (Appendix B: Figure A1). Figure 2 shows the scree plots of the principal components for each cohort. Each principal component represents a linear combination of the AE-level composite scores, designed to be orthogonal to each other. Eigenvalues reflect the amount of variance explained by each principal component, with the first eigenvalue accounting for 43%, 36%, and 39% of the variance in the lung, breast, and head and neck cohorts, respectively. Subsequent eigenvalues flattened substantially, as shown in Figure 2. The eigenvalue ratio (*R_i_*) was highest for the first component across cohorts (3.39 for lung, 4.38 for breast, and 4.42 for head and neck), supporting the retention of a single principal component.

#### 3.3.2. CFA Model for the Lung Cohort

**Figure A2:**
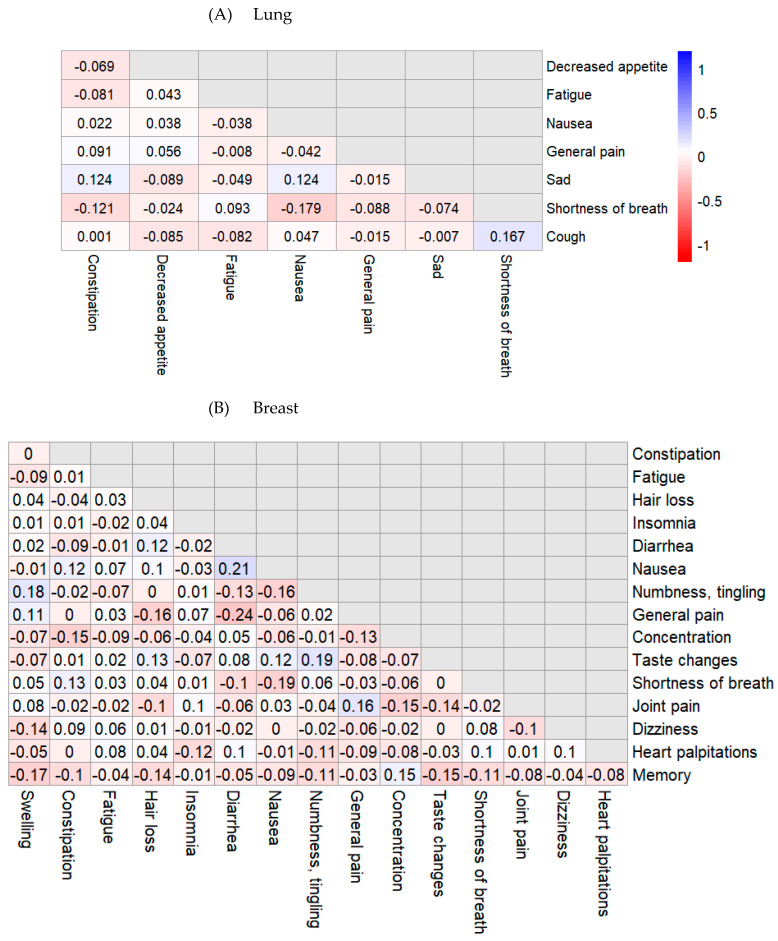

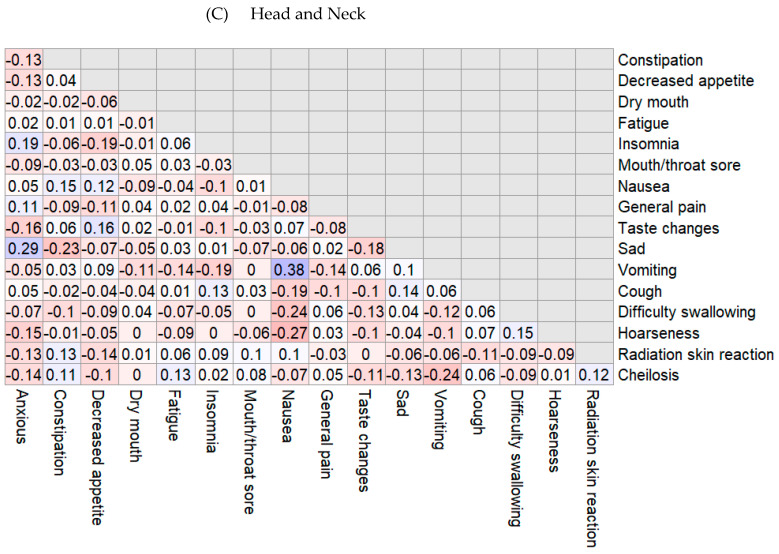
Residual Correlations from the CFA Model.

The CFA model demonstrated strong fit in the lung cohort: CFI = 0.99, TLI = 0.98, RMSEA = 0.067 (90% CI: 0.029–0.101), and SRMR = 0.072. The chi-square statistic was *χ*^2^(20) = 36.1, *p* = 0.02. Residual correlations ranged from −0.18 to 0.17 with a mean of −0.01 (Appendix C: Figure A2).

#### 3.3.3. CFA Model for the Breast Cohort

The CFA model in the breast cohort showed a mixed fit. While CFI = 0.98 and TLI = 0.97 indicated excellent fit, other indices demonstrated less than ideal fit, including RMSEA = 0.077 (90% CI: 0.066, 0.089), SRMR = 0.083, and *χ*^2^ (104) = 265.2, *p* < 0.001. Residual correlations ranged from −0.24 to 0.22 with a mean of −0.01 (Appendix C: Figure A2). A respecified model including residual correlations such as those between nausea and diarrhea or numbness/tingling and taste changes achieved better fit: CFI = 0.98 and TLI = 0.98, RMSEA = 0.068 (90% CI: 0.056–0.080), SRMR = 0.076, and *χ*^2^ (101) = 221.3, *p* < 0.001.

#### 3.3.4. CFA Model for the Head and Neck Cohort

For the head and neck cohort, the CFA model demonstrated excellent fit based on some indices, including CFI = 0.98 and TLI = 0.98, but others indicated less than ideal model fit, such as RMSEA = 0.086 (90% CI: 0.071, 0.101), SRMR = 0.098, and *χ*^2^ (119) = 245.9, *p* < 0.001. Residual correlations ranged from −0.27 to 0.38, with a mean of −0.02 (Appendix C: Figure A2). Incorporating residual correlations (e.g., between nausea and vomiting, anxious and sad) improved model fit: CFI = 0.99, TLI = 0.99, RMSEA = 0.059 (90% CI: 0.040–0.077), SRMR = 0.081, and *χ*^2^ (114) = 172.0, *p* = 0.001.

#### 3.3.5. CFA Factor Loadings Across Three Cohorts

The CFA factor loadings demonstrated the AE terms most strongly associated with the latent factor in each cohort (Table 2). In the lung cohort, fatigue, pain, decreased appetite, and shortness of breath exhibited the strongest relationships to the latent factor. For the breast cohort, fatigue, concentration, memory, and pain were most strongly correlated with the latent factor, whereas in the head/neck cohort, difficulty swallowing, dry mouth, taste changes, and decreased appetite exhibited the strongest association with the latent factor.

### 3.4. Reliability and Convergent Validity

**Figure 3:**
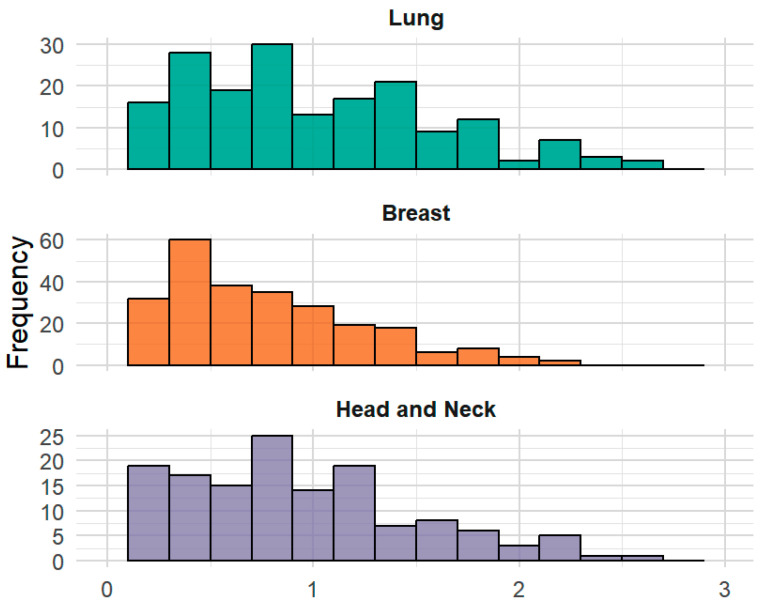
Distribution of Average Composite Score (ACS) Across 3 Disease Sites. Note. Because composite scores range from 0 to 3, the ACS also theoretically ranges from 0 to 3. However, in practice, the maximum score of 3 on ACS is rarely observed, as it is uncommon for individuals to have a score of 3 on composite scores across all AE terms measured. As a result, the ACS often does not reach its theoretical maximum, despite having a defined range of 0 to 3.

**Figure 4:**
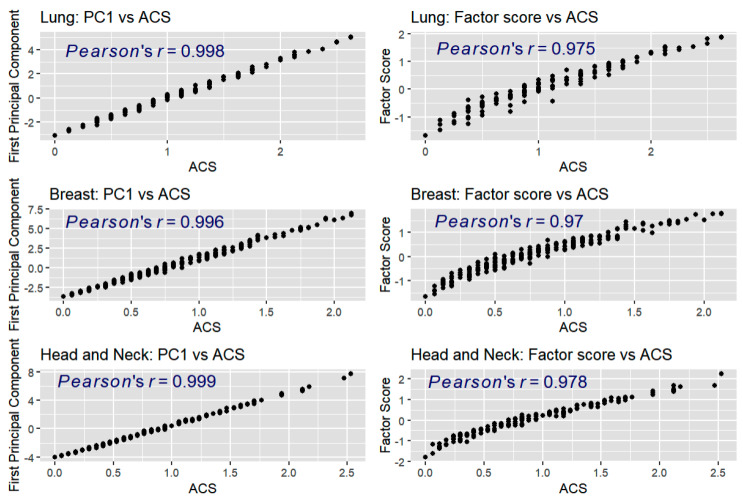
Scatter plots of composite averages vs. the scores derived from principal component analysis (PC1) and confirmatory factor analysis (CFA) across three cohorts. Note. Each cohort—Lung (**top** row), Breast (**middle** row), and Head and Neck (**bottom** row)—includes two plots: PC1 vs. ACS (**left**) and Factor score vs. ACS (**right**). High Pearson correlation coefficients (ranging from 0.97 to 0.999) indicate strong linear associations, supporting the validity of ACS as a summary indicator of symptomatic AE burden.

ACS ranged from 0 to 2.63, with a mean (SD) of 0.99 (0.60) in the lung cohort; from 0 to 2.13 with a mean (SD) of 0.75 (0.48) in the breast cohort; and from 0 to 2.53, with a mean (SD) of 0.90 (0.57) in the head/neck cohort (Figure 3, Table 1). McDonald’s *ω* were 0.81, 0.88, and 0.91, respectively. ACS correlated strongly with factor scores from the CFA models (*r* = 0.975, 0.969, 0.977) demonstrating evidence for structural validity in assessing the latent construct of symptomatic AE burden. ACS also correlated strongly with the first principal component from the principal component analysis (0.998, 0.996, and 0.999) (Figure 4). Pearson correlations between the ACS and the EORTC QLQ-C30 summary score were 0.85 (lung), 0.84 (breast), and 0.83 (head/neck), respectively, supporting strong convergent validity.

### 3.5. Impact of Reducing the Number of Symptom Terms on Reliability and Validity

Across all steps of sequential reduction in the number of symptom terms in both the breast and the head/neck cohorts, the eigenvalue ratio consistently supported retention of a single factor/component. As terms were sequentially removed in order of least to most frequently endorsed by patients in prior studies (Table 3), the percentage of variance explained by PC1 progressively increased. In the breast cohort, McDonald’s ω gradually decreased from 0.88 to 0.69 when only four of the original 16 terms remained. In the head and neck cohort, McDonald’s ω decreased from 0.91 to 0.83 when four of the original 17 terms remained, with a drop to 0.74 when “decreased appetite” was excluded. When terms were removed sequentially in order of smallest to largest factor loadings (Appendix D: Table A2), McDonald’s *ω* decreased less sharply, because the remaining terms were more homogeneous when factor loadings guided AE term removal, highlighting the differing impacts of these two strategies for term removal.

Despite these reductions in reliability, the ACS computed using three or more symptom terms remained highly correlated with the ACS from the full set (all correlations *r* ≥ 0.87 in the breast cohort and *r* ≥ 0.91 in the head and neck cohort) (Table 3). Pearson’s correlation with the QLQ-C30 summary score decreased slightly, from 0.85 to 0.76 (16-term to 3-term set) in the breast cohort and from 0.83 to 0.75 (17-term to 3-term set) in the head and neck cohort. The fit indices, especially CFI and TLI, for CFA models remained above 0.95 across all steps of sequentially removing the AE term, and SRMR values were similarly robust below 0.080 allowing for residual correlations, indicating excellent relative fit even with reduced terms. These results suggest that the ACS retains favorable reliability and structural validity summarizing symptomatic AE burden at a given timepoint, even when fewer AE-level terms are included.

### 3.6. Comparing Sensitivity and Interpretability of the ACS

**Figure 5:**
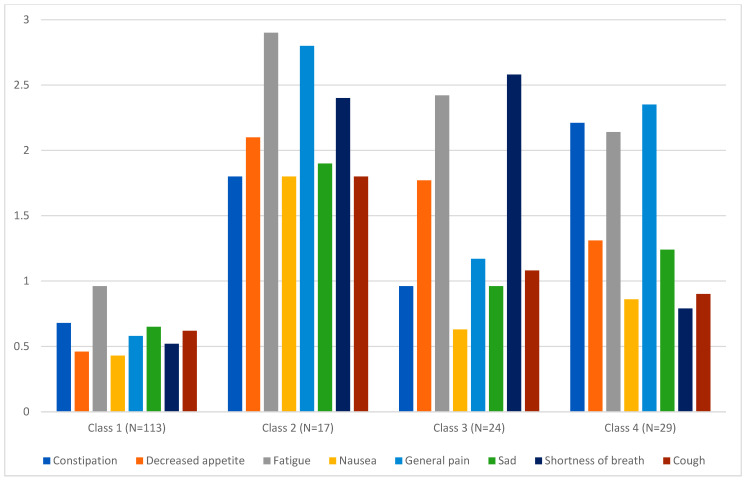
Symptomatic AE profiles across four latent classes in the lung cohort (*N* = 183). Note. Four distinct symptomatic AE profiles were observed in the lung cohort. Each latent profile was characterized by a distinct symptom expression pattern. Class-specific mean AE-level composite scores (range 0–3) are depicted on the Y axis. While all classes exhibited a distinct symptomatic AE profile, only latent classes 1 and 2 (high on all symptoms versus low on all symptoms) demonstrated significantly different mean ACS scores. The mean ACS scores for classes 3 and 4 were nearly identical, yet the profiles themselves were qualitatively distinct. Individuals in latent class 3 were experiencing significant appetite loss, fatigue and shortness of breath, whereas those in latent class 4 were experiencing significant constipation, fatigue and pain. The mean (SD) of ACS was 0.6 (0.3) in class 1 (low on all symptoms), 2.1 (0.3) in class 2 (high on all symptoms), 1.4 (0.3) in class 3 (elevated appetite loss, fatigue, and shortness of breath), and 1.5 (0.2) in class 4 (elevated constipation, fatigue, and pain).

We used LPA to determine if ACS values distinguish respondents with clinically distinct symptomatic AE profiles, modeling individual AE composite scores in the lung cohort as an example. The 4-profile solution was selected due to a lower BIC (3635.6), higher entropy (0.91), high average posterior probability (0.94), and all classes having at least ten individuals (Table 4). Figure 5 displays the class-specific mean AE-level composite scores for each of the eight AE terms across the four latent classes. The mean ACS was 0.6 in class 1, 2.1 in class 2, 1.4 in class 3, and 1.5 in class 4. Notably, latent profile classes with similar ACS values exhibited distinct AE profiles. Class 3 (*N* = 24) and Class 4 (*N* = 29), both had an ACS of approximately 1.5. However, Class 3 reported more shortness of breath (2.6 vs. 0.8), while class 4 reports more pain (2.4 vs. 1.2) and constipation (2.2 vs. 1.0). Taken together, these observations demonstrate that ACS scores are sensitive to differences in overall symptomatic AE burden, and at the same time, respondents with the same level of overall AE burden can exhibit a different profile of symptomatic AEs (predominant constipation, fatigue and general pain versus predominant decreased appetite, fatigue and shortness of breath). These observations underscore the importance of characterizing the dimensional profile of symptomatic AEs in addition to presenting a summary metric of symptomatic AE burden.

## 4. Discussion

Patient-reported outcomes have increasingly been recognized for their potential to expand the assessment of treatment tolerability to include the patient’s lived experience [41,42]. Measures such as the PRO-CTCAE Item Library including 124 items representing 78 symptomatic toxicities offer promising approaches to systematically capture a wide range of symptomatic AEs experienced during and following cancer treatment. Recently, single-item global measures of the burden of treatment side effects —such as the GP5 item from the Functional Assessment of Chronic Illness Therapy (FACIT) [43,44] and the Q168 item from the EORTC library [45]—have gained more attention reflecting growing recognition of the value of summary metrics that capture overall side effect impact. In alignment with this trend, the U.S. Food and Drug Administration has recommended the development and inclusion of global side effect items within existing PRO libraries [46].

However, single items that assess the global burden of treatment-related side effects may be of limited utility if the goal of a study is to understand the tolerability of a regimen, since a single global item alone does not distinguish the full spectrum of the symptomatic adverse events experienced by the patient (e.g., fatigue, pain, constipation, etc.) or their frequency, severity, or interference with usual or daily activities. A single item asking globally about treatment side effects may also be challenging to interpret prior to treatment initiation as the item phrasing presupposes exposure to therapy [47]. This is particularly relevant in oncology, where patients often present with substantial symptom burden at baseline [48], and distinguishing disease-related symptoms from treatment-related side effects can be challenging from patient perspectives. In terms of PRO-CTCAE, it enables baseline assessment as it does not attribute AE experiences to treatment, but its multi-item structure without a mechanism for generating an overall score produces a large volume of data to analyze, which poses a challenge especially when multiple comparisons are involved. As such, the availability of a psychometrically robust summary indicator greatly facilitates longitudinal analyses and simplifies data interpretation.

### 4.1. ACS as a Summary Metric for Symptomatic AE Burden

The PRO-CTCAE ACS offers a potential solution to these challenges by providing a summary score that is appropriate for baseline assessment and by addressing analytical limitations commonly associated with ordinal data [47]. This study is, to our knowledge, the first to propose the ACS as a cross-sectional summary metric of symptomatic AE burden, and to investigate its reliability, validity and interpretability. The strong fit of a one-factor model provides compelling justification for using the ACS as an estimate of the latent variable representing the overall burden of symptomatic AEs, both for the full and reduced set of terms. Additionally, the strong correlation with the validated QLQ-C30 summary score provides evidence of convergent validity. Furthermore, the AEs that contributed most significantly to the overall construct, as demonstrated by the factor loadings, varied across cohorts. This observation underscores the importance of capturing symptomatic AE terms that are most salient to the disease and treatment context.

An essential finding in our study was that while the PRO-CTCAE ACS effectively quantifies the cross-sectional burden of symptomatic AEs, it does not capture the specific profile of symptomatic toxicities as was shown by the LPA. Patients with similar ACS values exhibited clinically distinct symptom experiences, highlighting the limitations of summary scores and the complementary interpretive value of both summary scores and the characteristics of the symptom profile. As such, relying solely on the ACS could obscure insights into individual symptomatic AEs that are crucial for a complete understanding of the patient experience and for precision in tailoring interventions to improve tolerability. Thus, presenting detailed profile of symptomatic AEs in addition to a concise summary metric remains essential for a comprehensive understanding of treatment-emergent toxicities.

### 4.2. Potential Applications of ACS in Research and Clinical Settings

An important potential application of the ACS is to quantify and compare overall symptomatic AE burden between treatment arms or across timepoints. Beyond individual trials, the ACS could also support cross-trial comparisons, provided that studies administer an overlapping set of PRO-CTCAE symptom terms. In such cases, establishing consensus on essential AE terms relevant to the disease and treatment context is essential. For healthcare professionals, ACS offers a concise, interpretable metric that can aid in identifying patients with emerging treatment intolerability and guiding timely pre-emptive supportive care interventions. For patients and their clinicians, the ACS—especially when presented alongside the specific symptomatic AE profile (for example, pain, fatigue and sleep disturbance versus shortness of breath, appetite loss and constipation) can strengthen communication, improve shared decision-making and better target the delivery of supportive care strategies and self-management support.

### 4.3. Strengths, Limitations and Methodological Considerations for Future Research

The strengths of this study include a diverse sample encompassing various treatment regimens across nine U.S. cancer centers and community oncology practices, an in-depth focus into three tumor types, inclusion of 27 out of 78 PRO-CTCAE terms from the Item Library—each represented in one or more disease groups—and a wide range of complementary, methodologically robust analyses such as factor analyses and latent profile analyses. This study focused on PRO-CTCAE terms based on three attributes and did not include binary “presence” (yes/no) items. A further caveat is that our findings may not generalize to other PRO-CTCAE item subsets or other cancer sites, and replication studies are warranted to increase confidence in these findings.

Factor analyses were used in this study to assess dimensionality, as it is a well-established approach. However, factor scores imply a reflective model, where the burden of symptomatic AEs is assumed to causally influence responses to PRO-CTCAE items. Alternatively, a formative model may be an equally suitable approach to examine the validity of the ACS, where observed AE-level composite scores influence the latent construct of overall symptomatic AE burden. Complementing the latent variable framework, network psychometric modeling offers an alternative framework by conceptualizing covariance as arising from pairwise interactions between variables in a network structure [49]. Preliminary work suggests that in complex networks, sum score approaches like the ACS can be useful to assess the overall state of the network, even without strictly adhering to unidimensionality assumptions [19]. Future research could explore alternative statistical modeling approaches that might explain the data as well as, or perhaps better than, factor analysis [50].

## 5. Conclusions

In conclusion, the average composite score offers a psychometrically sound and easily calculated summary metric of AE burden. These favorable measurement properties of the ACS are maintained even when fewer AE-level composite scores are included in the ACS calculation. This study focused on validating the ACS based on the internal structure of the PRO-CTCAE data. Future research should explore additional aspects of ACS measurement properties including test–retest reliability, responsiveness to change, and ability to distinguish known-groups. Our exploration of sensitivity and interpretability using LPA should be replicated in other samples to determine whether our observations about the importance of characterizing both the overall burden and the profile of AEs are replicated in different subpopulations based on treatment type. Taken together, our results demonstrate that the ACS offers an intuitive, valid and easily calculated summary metric for use in clinical trials when interpreted alongside the dimensional AE profile.

## Figures and Tables

**Table 1:** Participant characteristics at study enrollment.

	Lung (*N* = 183)	Breast (*N* = 260)	Head/Neck (*N* = 146)
**Age at enrollment**			
Median (IQR)	62 (54, 69)	53 (47, 63)	58 (50, 66)
Range	26, 88	26, 91	20, 85
**Gender**, *n* (%)			
Female	99 (54.1%)	256 (98.5%)	33 (22.6)
Male	84 (45.9%)	4 (1.5%)	113 (77.4)
**Race**, *n* (%)			
White	148 (80.9%)	168 (64.6%)	115 (78.8%)
Black or African American	23 (12.6%)	66 (25.4%)	25 (17.1%)
Native Hawaiian, Other Pacific Islander	1 (0.5%)	0 (0%)	1 (0.7%)
Asian	9 (4.9%)	23 (8.8%)	5 (3.4%)
American Indian	1 (0.5%)	0 (0%)	0 (0%)
Multiple races reported	0 (0%)	0 (0%)	0 (0%)
Unknown	1 (0.5%)	3 (1.2%)	0 (0%)
**Ethnicity**, *n* (%)			
Hispanic/Latino	8 (4.4%)	21 (8.1%)	8 (5.5%)
Non-Hispanic	173 (94.5%)	225 (86.5%)	136 (93.2%)
Unknown/Not reported	2 (1.1%)	14 (5.4%)	2 (1.4%)
**Education level**, *n* (%)			
Less than high school	16 (8.7%)	12 (4.6%)	8 (5.5%)
High school or GED	52 (28.4%)	46 (17.7%)	33 (22.6%)
Some college	37 (20.2%)	66 (25.4%)	29 (19.9%)
College graduate or more	77 (42.1%)	134 (51.5%)	74 (50.7%)
Missing	0 (0.0%)	2 (0.8%)	0 (0.0%)
**ECOG PS (Visit 1)**, *n* (%)			
ECOG 0–1	140 (76.5%)	244 (93.8%)	117 (80.1%)
ECOG 2–4	43 (23.5%)	16 (6.2%)	29 (19.9%)
**Treatment (Visit 1)**, *n* (%)			
Radiation therapy in past 2 weeks	80 (43.7%)	89 (34.2%)	136 (93.2%)
Surgery in past 2 weeks	7 (3.8%)	6 (2.3%)	16 (11.0%)
Chemotherapy in past 2 weeks	136 (74.3%)	104 (40.0%)	102 (69.9%)
**Average Composite Scores**			
Mean (SD)	0.99 (0.60)	0.75 (0.48)	0.90 (0.57)
Median (IQR)	0.88 (0.50, 1.38)	0.69 (0.38, 1.06)	0.82 (0.43, 1.24)
Range	0, 2.63	0, 2.13	0, 2.53

**Table 2:** Factor loadings from the final CFA models, ordered by magnitude.

	Indicator	Unstandardized Estimate	Standard Error	*Z* Statistic	*p*-Value	Standardized Estimate
Lung	Fatigue	1	-	-	-	0.87
	General pain	0.853	0.064	13.42	<0.001	0.741
	Decreased appetite	0.742	0.071	10.41	<0.001	0.645
	Shortness of breath	0.739	0.069	10.724	<0.001	0.643
	Nausea	0.717	0.074	9.683	<0.001	0.624
	Sad	0.69	0.064	10.819	<0.001	0.6
	Constipation	0.641	0.079	8.067	<0.001	0.558
	Cough	0.535	0.077	6.955	<0.001	0.466
Breast	Fatigue	1.000	-	-	-	0.844
	Concentration	0.980	0.041	23.75	<0.001	0.827
	Memory	0.955	0.041	23.19	<0.001	0.806
	General pain	0.907	0.039	23.11	<0.001	0.766
	Joint pain	0.875	0.042	21.03	<0.001	0.739
	Dizziness	0.808	0.056	14.31	<0.001	0.682
	Nausea	0.754	0.052	14.57	<0.001	0.636
	Shortness of breath	0.722	0.061	11.79	<0.001	0.609
	Taste changes	0.694	0.063	10.95	<0.001	0.586
	Heart palpitations	0.689	0.056	12.41	<0.001	0.582
	Insomnia	0.683	0.054	12.75	<0.001	0.577
	Swelling	0.663	0.065	10.12	<0.001	0.560
	Numbness and tingling	0.630	0.064	9.89	<0.001	0.531
	Hair loss	0.564	0.070	8.02	<0.001	0.476
	Constipation	0.529	0.072	7.33	<0.001	0.446
	Diarrhea	0.373	0.079	4.72	<0.001	0.315
Head and Neck	Difficulty swallowing	1.000	-	-	-	0.877
	Dry mouth	0.926	0.044	20.93	<0.001	0.812
	Taste changes	0.925	0.043	21.45	<0.001	0.812
	Decreased Appetite	0.908	0.048	18.81	<0.001	0.796
	Mouth/throat sore	0.845	0.054	15.73	<0.001	0.741
	General pain	0.839	0.044	19.17	<0.001	0.736
	Hoarseness	0.822	0.060	13.61	<0.001	0.721
	Fatigue	0.800	0.063	12.65	<0.001	0.702
	Radiation skin reaction	0.793	0.080	9.86	<0.001	0.696
	Cheilosis	0.764	0.072	10.65	<0.001	0.670
	Cough	0.758	0.063	11.97	<0.001	0.665
	Nausea	0.696	0.070	9.96	<0.001	0.611
	Anxious	0.610	0.072	8.49	<0.001	0.535
	Constipation	0.606	0.081	7.51	<0.001	0.531
	Sad	0.590	0.077	7.64	<0.001	0.517
	Insomnia	0.583	0.073	7.97	<0.001	0.512
	Vomiting	0.577	0.088	6.55	<0.001	0.506

**Table 3:** Impact on model fit and validity metrics of sequential term removal based on patient ratings of prevalence and importance from prior mixed-methods studies [28,30].

(a) Breast Cohort												
Sequentially Removed Terms	Number of Remaining Terms	PC1 Variance (%)	McDonald’s *ω*	CFA Fit Indices					Pearson’s *r* Between ACS from the Reduced Set and			
				*χ*^2^(*df*), *p*-Value	CFI	TLI	RMSEA (90% CI)	SRMR	PC1 ^1^	CFA Factor Score ^1^	QLQ-C30 Summary	ACS from the Full Set
**Breast cohort**												
Swelling	15	37	0.87	124.9 (74), <0.001	0.988	0.986	0.052 (0.035, 0.067)	0.067	0.996	0.974	0.85	0.995
Memory	14	36	0.86	136.4 (75), <0.001	0.986	0.983	0.056 (0.041, 0.071)	0.070	0.996	0.975	0.84	0.992
Heart palpitations	13	37	0.85	107.0 (62), <0.001	0.988	0.985	0.053 (0.035, 0.070)	0.067	0.996	0.974	0.84	0.990
Shortness of breath	12	37	0.84	101.3 (52), <0.001	0.986	0.982	0.060 (0.043, 0.078)	0.070	0.996	0.960	0.83	0.985
Dizziness	11	38	0.83	91.0 (42), <0.001	0.983	0.978	0.067 (0.048, 0.086)	0.072	0.996	0.974	0.82	0.981
Taste changes	10	38	0.82	64.2 (34), <0.001	0.988	0.984	0.059 (0.036, 0.080)	0.068	0.995	0.972	0.83	0.975
Constipation	9	41	0.83	45.3 (25), 0.008	0.991	0.987	0.056 (0.028, 0.82)	0.063	0.997	0.974	0.81	0.962
Diarrhea	8	45	0.83	37.4 (20), 0.010	0.992	0.989	0.058 (0.028, 0.086)	0.061	0.998	0.978	0.82	0.958
General pain	7	44	0.79	17.9 (14), 0.213	0.997	0.995	0.033 (0.000, 0.072)	0.050	0.998	0.976	0.80	0.951
Concentration	6	45	0.76	12.7 (9), 0.177	0.996	0.993	0.040 (0.000, 0.086)	0.050	0.998	0.974	0.78	0.932
Joint pain	5	45	0.70	5.6 (5), 0.352	0.999	0.997	0.021 (0.000, 0.091)	0.039	0.998	0.954	0.74	0.907
Hair loss	4	50	0.69	4.4 (2), 0.114	0.992	0.977	0.067 (0.000, 0.156)	0.042	0.998	0.932	0.79	0.906
Insomnia	3	56	-	- ^2^	-	-	-	-	0.991	-	0.76	0.872
^1^ These were calculated within the reduced set for each step. ^2^ For the final row, after removing ‘insomnia,’ three terms (fatigue, numbness & tingling, and nausea) remained in the set. McDonald’s *ω* or the CFA factor score could not be calculated for the one-factor model with three terms due to a negative residual variance for ‘fatigue’ in this model. Note. PC1 = First Principal Component. McDonald’s omega = Reliability coefficient estimating internal consistency based on factor loadings; values ≥ 0.70 are generally considered acceptable. CFI = Comparative Fit Index and TLI = Tucker–Lewis Index; values ≥ 0.95 indicate excellent model fit. RMSEA = Root Mean Square Error of Approximation; values ≤ 0.06 suggest good fit. SRMR = Standardized Root Mean Square Residual; values ≤ 0.08 are considered acceptable.												
**(b) Head and Neck Cohort**												
**Sequentially Removed Terms**	**Number of Remaining Terms**	**PC1 Variance (%)**	**McDonald’s *ω***	**CFA Fit Indices**					**Pearson’s *r* Between ACS from the Reduced Set and**			
				***χ*^2^(*df*), *p*-Value**	**CFI**	**TLI**	**RMSEA** **(90% CI)**	**SRMR**	**PC1 ^1^**	**CFA Factor Score ^1^**	**QLQ-C30 Summary**	**ACS from the Full Set**
**Head and neck cohort**												
Anxious	16	41	0.91	142.0 (100), 0.004	0.992	0.991	0.054 (0.031,0.073)	0.078	0.999	0.979	0.83	0.997
Constipation	15	42	0.91	127.3 (87), 0.003	0.992	0.990	0.058 (0.035,0.078)	0.076	0.999	0.979	0.82	0.993
Vomiting	14	44	0.91	118.8 (76), 0.001	0.991	0.990	0.062 (0.039,0.083)	0.076	0.999	0.981	0.81	0.991
Cracking at the corners of the mouth	13	45	0.90	97.3 (63), 0.004	0.992	0.990	0.061 (0.036, 0.084)	0.073	0.999	0.981	0.81	0.989
Radiation skin reaction	12	46	0.90	88.9 (53), 0.001	0.991	0.989	0.068 (0.042, 0.093)	0.075	0.999	0.981	0.81	0.986
Sad	11	48	0.90	78.6 (43), 0.001	0.990	0.988	0.076 (0.048, 0.102)	0.075	0.999	0.981	0.80	0.982
Insomnia	10	51	0.90	67.2 (34), 0.001	0.991	0.988	0.082 (0.053, 0.111)	0.073	0.999	0.983	0.79	0.976
Cough	9	53	0.89	51.5 (25), 0.001	0.992	0.988	0.085 (0.052, 0.119)	0.065	0.999	0.984	0.78	0.969
Mouth/throat sore	8	54	0.88	48.2 (18), <0.001	0.989	0.982	0.108 (0.071, 0.145)	0.071	0.999	0.978	0.79	0.961
Nausea	7	58	0.88	43.2 (14), <0.001	0.988	0.982	0.120 (0.080, 0.161)	0.073	0.999	0.987	0.77	0.947
General pain	6	60	0.87	36.2 (9), <0.001	0.986	0.976	0.144 (0.097, 0.195)	0.076	0.999	0.989	0.74	0.934
Hoarseness	5	63	0.86	14.9 (5), 0.011	0.993	0.985	0.117 (0.051, 0.188)	0.055	0.999	0.990	0.75	0.928
Dry mouth	4	65	0.83	2.1 (2), 0.349	1.000	1.000	0.019 (0.000, 0.167)	0.031	0.999	0.981	0.77	0.910
Decreased Appetite	3	65	0.74	- ^2^	-	-	-	-	0.999	0.994	0.75	0.909
^1^ These were calculated within the reduced set for each step. ^2^ For the final row, after removing “decreased appetite,” three terms (difficulty swallowing, taste changes, and fatigue) remained in the set. CFA model fit indices could not be calculated for the 3-term model, because it is just-identified. Note. PC1 = First Principal Component. McDonald’s omega = Reliability coefficient estimating internal consistency based on factor loadings; values ≥ 0.70 are generally considered acceptable. CFI = Comparative Fit Index and TLI = Tucker–Lewis Index; values ≥ 0.95 indicate excellent model fit. RMSEA = Root Mean Square Error of Approximation; values ≤ 0.06 suggest good fit. SRMR = Standardized Root Mean Square Residual; values ≤ 0.08 are considered acceptable.												

**Table 4:** Comparison of fit indices and class distributions for 1 through 6 latent profile classes (lung cohort, *n* = 183).

Number of Classes	AIC	BIC	Entropy †	Average Posterior Probability (min-max) ‡	LMR Likelihood Ratio Test ▪	BLRT §	Number of People in Each Latent Class					
							1	2	3	4	5	6
1	3912.9	3964.3	NA	1	NA	NA	183					
2	3576.8	3657.0	0.92	0.976 (0.970–0.982)	Significant	Significant	124	59				
3	3545.7	3654.9	0.89	0.943 (0.909–0.970)	Not significant	Significant	118	54	11			
4 *	**3497.6**	**3635.6**	**0.91**	**0.939 (0.907–0.969)**	Not significant	Significant	**113**	**17**	**24**	**29**		
5	3472.7	3639.6	0.92	0.957 (0.929–0.995)	Not significant	Significant	6	30	111	25	11	
6	3376.9	3572.7	0.97	0.811 (0.000–1.000)	◊	◊	0	75	34	20	26	28

Akaike Information Criterion (AIC) and Bayesian Information Criterion (BIC): Lower AIC or BIC indicates better fit. † Entropy value ranges from 0 to 1 with a higher value indicating more accurate classification into latent classes. ‡ Average of the probabilities that the individual belongs to the assigned latent profile class. Posterior probabilities provide a way to understand the uncertainty associated with assigning individuals to classes, as individuals are typically assigned to the class with the highest posterior probability, also known as the modal class. As such higher average posterior probabilities reflect more accurate individual-level classification. ▪ Lo, Mendell, and Rubin likelihood ratio test. A significant result indicates a better fit for the tested model (K). § Bootstrapped likelihood ratio test. A significant result indicates a better fit for the tested model (K) compared to the model with one fewer class (K-1). NA = not applicable. ◊ Likelihood ratio test could not be computed for the 6-class model because the estimation of the 6-class model with 5-class did not converge. * 4-profile class solution selected based on low BIC, high entropy, high average posterior probability, acceptable class sizes, and substantive interpretability.

## Data Availability

Restrictions apply to the availability of these data. Data were obtained from the U.S. National Cancer Institute and are available, with the permission of the National Cancer Institute, from SAM (Sandra.mitchell@nih.gov).

## Abbreviations

The following abbreviations are used in this manuscript:

AE	Adverse event
PRO-CTCAE	Patient-Reported Outcome version of the Common Terminology Criteria for Adverse Events
ACS	Average composite score
CFA	Confirmatory factor analysis
PCA	Principal component analysis
LPA	Latent profile analysis
EORTC QLQ-C30	European Organisation for Research and Treatment of Cancer Core Quality of Life questionnaire
ECOG PS	Eastern Cooperative Oncology Group Performance Status
CFI	Comparative fit index
TLI	Tucker–Lewis index
RMSEA	Root mean square error of approximation
SRMR	Standardized root mean square residual
FIML	Full information maximum likelihood
BIC	Bayesian Information Criterion

## Appendix A

**Table A1:** PRO-CTCAE Terms Identified as Relevant for Three Cancer types.

PRO-CTCAE Symptomatic AE Terms	Lung	Breast	Head/Neck
Dry mouth			X
Difficulty swallowing			X
Mouth/throat sores			X
Cracking at the corners of the mouth			X
Hoarseness			X
Taste changes		X	X
Decreased appetite	X		X
Nausea	X	X	X
Vomiting			X
Constipation	X	X	X
Diarrhea		X	
Shortness of breath	X	X	
Cough	X		X
Swelling		X	
Heart palpitations		X	
Hair loss		X	
Radiation skin reaction			X
Numbness & tingling		X	
Dizziness		X	
Concentration		X	
Memory		X	
General Pain	X	X	X
Joint pain		X	
Insomnia		X	X
Fatigue	X	X	X
Anxious			X
Sad	X		X

Note. Lung cancer terms as identified in Veldhuijzen et al. [32], breast cancer terms as identified in Günther et al. [28], and head/neck terms as identified in Sandler et al. [30] were used in this study.

## Appendix D

**Table A2:** Impact on Model Fit and Validity Metrics of Sequential Term Removal Based on Factor Loadings based on patient ratings of prevalence and importance from prior mixed-methods studies [28,30].

(a) Breast Cohort												
Sequentially Removed Terms	Number of Remaining Terms	PC1 Variance (%)	McDonald’s *ω*	CFA Fit Indices					Pearson’s *r* Between ACS from the Reduced Set and			
				*χ*^2^(*df*), *p*-Value	CFI	TLI	RMSEA (90% CI)	SRMR	PC1^1^	CFA Factor Score ^1^	QLQ-C30 Summary	ACS from the Full Set
**Breast cohort**												
Diarrhea	15	38	0.88	207.7 (89), <0.001	0.982	0.978	0.072 (0.059, 0.085)	0.076	0.996	0.970	0.85	0.997
Constipation	14	39	0.88	191.3 (76), <0.001	0.981	0.978	0.077 (0.063, 0.090)	0.077	0.997	0.972	0.84	0.991
Hair loss	13	41	0.88	150.5 (62), <0.001	0.985	0.981	0.074 (0.059, 0.089)	0.070	0.998	0.973	0.85	0.985
Numbness and tingling	12	42	0.87	140.0 (52), <0.001	0.984	0.979	0.081 (0.065, 0.097)	0.072	0.997	0.973	0.85	0.982
Swelling	11	44	0.87	129.3 (44), <0.001	0.983	0.979	0.087 (0.069, 0.104)	0.074	0.998	0.975	0.86	0.972
Insomnia	10	45	0.87	85.1 (34), <0.001	0.989	0.985	0.076 (0.056, 0.097)	0.068	0.998	0.971	0.85	0.964
Heart palpitations	9	45	0.86	101.3 (27), <0.001	0.981	0.975	0.103 (0.082, 0.125)	0.078	0.991	0.973	0.85	0.961
Taste changes	8	50	0.86	83.3 (20), <0.001	0.982	0.975	0.111 (0.087, 0.136)	0.078	0.999	0.977	0.86	0.948
Shortness of breath	7	53	0.86	73.5 (14), <0.001	0.982	0.973	0.128 (0.100, 0.158)	0.077	0.999	0.979	0.85	0.933
Nausea	6	57	0.85	47.9 (8), <0.001	0.986	0.974	0.139 (0.102, 0.178)	0.075	0.998	0.982	0.84	0.919
Dizziness	5	62	0.85	36.0 (4), <0.001	0.987	0.967	0.176 (0.126, 0.231)	0.078	0.999	0.978	0.83	0.906
Joint pain	4	66	0.81	19.0 (2), <0.001	0.990	0.971	0.181 (0.113, 0.259)	0.064	0.997	0.974	0.81	0.886
General pain	3	72	0.80	- ^2^	-	-	-	-	0.998	0.977	0.79	0.849
^1^ These were calculated within the reduced set for each step. ^2^ For the final row, after removing “pain,” three terms (fatigue, concentration, and memory) remained in the set. CFA model fit indices could not be calculated for the 3-term model, because it is just-identified. Note. PC1 = First Principal Component. McDonald’s omega = Reliability coefficient estimating internal consistency based on factor loadings; values ≥ 0.70 are generally considered acceptable. CFI = Comparative Fit Index and TLI = Tucker-Lewis Index; values ≥ 0.95 indicate excellent model fit. RMSEA = Root Mean Square Error of Approximation; values ≤ 0.06 suggest good fit. SRMR = Standardized Root Mean Square Residual; values ≤ 0.08 are considered acceptable.												
**(b) Head and Neck cohort**												
**Sequentially Removed Terms**	**Number of Remaining Terms**	**PC1 Variance (%)**	**McDonald’s *ω***	**CFA Fit Indices**					**Pearson’s *r* Between ACS from the Reduced Set and**			
				***χ*^2^(*df*), *p*-Value**	**CFI**	**TLI**	**RMSEA (90% CI)**	**SRMR**	**PC1^1^**	**CFA Factor Score ^1^**	**QLQ-C30 Summary**	**ACS from the Full Set**
**Head and neck cohort**												
Vomiting	16	41	0.91	145.6 (99), 0.002	0.992	0.990	0.057 (0.036, 0.072)	0.076	0.999	0.979	0.83	0.999
Insomnia	15	42	0.91	122.2 (85), 0.005	0.993	0.991	0.055 (0.031, 0.076)	0.072	0.999	0.979	0.82	0.996
Sad	14	44	0.90	110.5 (74), 0.004	0.993	0.991	0.058 (0.034, 0.080)	0.073	0.999	0.979	0.82	0.992
Constipation	13	45	0.90	98.7 (62), 0.002	0.992	0.990	0.064 (0.038, 0.087)	0.072	0.999	0.980	0.81	0.989
Anxious	12	47	0.90	83.4 (51), 0.003	0.993	0.990	0.066 (0.039, 0.091)	0.069	0.999	0.982	0.79	0.981
Nausea	11	49	0.90	74.8 (44), 0.003	0.992	0.991	0.070 (0.041, 0.096)	0.069	0.999	0.982	0.77	0.972
Cough	10	51	0.90	67.1 (35), 0.001	0.991	0.989	0.080 (0.050, 0.108)	0.070	0.999	0.983	0.77	0.966
Cracking at the corners of the mouth	9	53	0.89	53.3 (27), 0.002	0.992	0.989	0.082 (0.049, 0.114)	0.067	0.999	0.978	0.76	0.963
Radiation skin reaction	8	57	0.89	46.0 (20), 0.001	0.991	0.988	0.095 (0.059, 0.131)	0.066	0.999	0.980	0.76	0.957
Fatigue	7	59	0.88	42.2 (14), <0.001	0.989	0.983	0.118 (0.078, 0.160)	0.071	0.999	0.986	0.72	0.944
Hoarseness	6	61	0.87	26.8 (9), 0.002	0.991	0.984	0.117 (0.067, 0.169)	0.066	0.999	0.980	0.73	0.942
General pain	5	64	0.86	17.9 (5), 0.003	0.991	0.982	0.133 (0.070, 0.203)	0.061	0.999	0.990	0.69	0.925
Mouth/throat sore	4	69	0.85	14.6 (2), 0.001	0.988	0.965	0.209 (0.117, 0.315)	0.067	0.999	0.993	0.70	0.904
Decreased Appetite	3	73	0.81	- ^2^	-	-	-	-	0.999	0.970	0.65	0.892
^1^ These were calculated within the reduced set for each step. ^2^ For the final row, after removing “decreased appetite,” three terms (difficulty swallowing, dry mouth, and taste changes) remained in the set. CFA model fit indices could not be calculated for the 3-term model, because it is just-identified. Note. PC1 = First Principal Component. McDonald’s omega = Reliability coefficient estimating internal consistency based on factor loadings; values ≥ 0.70 are generally considered acceptable. CFI = Comparative Fit Index and TLI = Tucker-Lewis Index; values ≥ 0.95 indicate excellent model fit. RMSEA = Root Mean Square Error of Approximation; values ≤ 0.06 suggest good fit. SRMR = Standardized Root Mean Square Residual; values ≤ 0.08 are considered acceptable.												
